# Experiences and needs of women in vulnerable situations receiving additional interventions in maternity care: a qualitative study

**DOI:** 10.1186/s12884-022-04847-0

**Published:** 2022-07-02

**Authors:** Esther I. Feijen-de Jong, Maria Dalmaijer, Relinde A. van der Stouwe, Danielle E. M. C. Jansen, J. Catja Warmelink

**Affiliations:** 1 University of Groningen, University Medical Center Groningen, Department of General Practice & Elderly Care Medicine, Groningen, The Netherlands; 2 Department of Midwifery Science AVAG, Amsterdam Public Health Research Institute, Amsterdam University Medical Centers (location Vumc), Van der Boechorststraat 7, 1081 BT Amsterdam, The Netherlands; 3 Midwifery Academy Amsterdam/Groningen, Dirk Huizingastraat 3-5, 9713 GL Groningen, The Netherlands

**Keywords:** Interventions, Health professionals, Pregnancy, Maternity care, Vulnerable, Stigma, Research on special populations

## Abstract

**Background:**

Tailoring an intervention to the needs and wishes of pregnant women in vulnerable situations (e.g., socioeconomic disadvantages) can reduce the risk of adverse outcomes and empower these women. A relatively high percentage of pregnant women in the North of the Netherlands are considered vulnerable to adverse pregnancy outcomes because of their low socioeconomic status and the intergenerational transmission of poverty. In order to improve perinatal and maternal health, next to standard prenatal care, various interventions for pregnant women in vulnerable situations have been developed. We do not know to what extent these additional interventions suit the needs of (pregnant) women. Therefore, the aim of this study is to gain insight into the experiences and needs of women in vulnerable situations who receive additional maternity care interventions in the Northern Netherlands.

**Methods:**

Qualitative research was performed. We used a phenomenological framework, which is geared towards understanding people’s experiences in the context of their everyday lives. In-depth semi-structured interviews were conducted with 17 pregnant women in vulnerable situations living in the Northern Netherlands. A thematic analysis was carried out.

**Results:**

We found three themes that reflect the experiences and needs of pregnant women in vulnerable situations in relation to the intervention they receive. These themes relate to the care provided by health professionals, to the impact of being offered an intervention, and to practical issues related to receiving an additional intervention. We found that the needs of pregnant women in vulnerable situations who received an additional maternity care intervention varied. This variation in needs was mainly related to practical issues. Women also expressed common needs, namely the desire to have control over their situation, the wish to receive tailor-made information about the intervention, and the wish for the intervention to be specifically tailored to their circumstances.

**Conclusions:**

Living in vulnerable situations and being offered additional care evoked diverse reactions and emotions from pregnant women. We recommend that health professionals ensure open and clear communication with women, that they ensure continuity of care and relationship-centered care, and that they become aware of the process of stigmatization of women in vulnerable situations.

## Background

Pregnant women in vulnerable situations often have diverse and complex needs. Briscoe et al. (page 2338, 2016) defined vulnerability as follows: “women are vulnerable when they experience ‘threat’ from a physical, psychological or social perspective, where ‘barriers’ and ‘reparative’ conditions influence the level of vulnerability” [1]. From this premise, it follows that becoming vulnerable involves a series of events that intertwine and create complexity for the woman and those providing maternity care [1]. Compared to non-vulnerable women, pregnant women in vulnerable situations have higher risks of perinatal mortality and morbidity [2–4]. Many of these risks can be linked to daily and cumulative stress factors, lower overall levels of social support, less diverse networks, and low socioeconomic status [1, 5–7].

In the Netherlands, many women in vulnerable situations - reflected by a low Social Economic Status (SES) - live in the three northern provinces [7, 8]. Across the Netherlands in its entirety, 25% of pregnant women can be designated as having a low SES. In the northern region, this percentage is 36% [8]. This group of women is mostly of Dutch origin and lives in rural areas. Poverty is transmitted intergenerationally within this segment of the Dutch population [9]. In addition, women of childbearing age in this region more often have unfavourable lifestyle characteristics than women in other parts of the Netherlands: they have a higher bodyweight (Groningen, Friesland), smoke more often (Drenthe), and are more likely to drink alcohol (Groningen, Drenthe) compared to the national average [8, 10, 11]. These figures indicate that a relatively large proportion of pregnant women in the Northern Netherlands have a relatively high risk of developing adverse pregnancy outcomes, such as pre-eclampsia, gestational diabetes, and congenital anomalies (due to obesity) [12–14], low birth weight and premature birth (due to smoking) [15], and miscarriage and perinatal mortality (due to alcohol) [16, 17].

In order to tailor care to the needs of pregnant women in vulnerable situations, many interventions have been developed at international, national, and regional level [18]. Sometimes, these interventions are incorporated into standard prenatal care (e.g. group prenatal care) provided by midwives and / or gynaecologists. In other cases, they are employed as additional programmes alongside standard care (e.g. additional care by community care workers in case of financial problems). There is only limited research available on the provision of additional interventions and the perceptions of pregnant women in vulnerable situations themselves about this offer. These studies show that women may feel stigmatized by this offer and may respond with anxiety and a sense of inadequacy [19]. Moreover, offering and implementing additional interventions to women in vulnerable situations may even lead to unintended harm, as they are not tailored to the unique environmental, cultural, economic, and health system situation of these women [20]. It may lead to refusal of the offer, which is often caused by differences in professional and lay perceptions of vulnerability and need [20]. It is also possible that women do not consider their circumstances as unusual and therefore do not consider themselves to be in need of support [21]. In addition, a lack of information about the interventions offered may lead to confusion and concern about being judged as a parent [21]. A key factor in the willingness to participate in a supportive intervention is establishing a trusting and non-judgmental relationship with the woman [19], where bridging the social gap between women and health professionals is a challenge [21]. Due to the limited research on this topic, specifically in a group of women of Dutch origin, it is important to explore whether interventions are in line with the experiences and needs of pregnant women in vulnerable situations themselves.

The current study aims to gain insight into the experiences and needs of women in vulnerable situations with regard to receiving additional maternity care interventions in the Northern Netherlands. The term “additional maternity care interventions” refers to the programs provided parallel to standard prenatal care to address various problems such as socioeconomic, financial, or psychosocial problems. The purpose of this study leads to our research question: ‘What are the experiences and needs of women in vulnerable situations living in the Northern Netherlands who receive additional maternity care interventions?’ This knowledge can be used to better tailor the interventions to the needs of these women.

## Methods

### Study design

In order to gain insight into the experiences and needs of women in vulnerable situations we used a phenomenological framework, an interpretivist paradigm, which is geared towards understanding people’s experiences in the context of their everyday lives. In-depth semi-structured interviews were conducted using a topic list (Table 1). The Standards for Reporting Qualitative Research checklist was used in preparation of this article. This study is part of a larger research project [22–24] that aims to improve the effectiveness and implementation of interventions for pregnant women in vulnerable situations from the perspective of these women themselves as well as from the perspective of healthcare professionals.

**Table 1:** Interview questions formulated on the basis of the topics on the list

•How would you introduce yourself in terms of, for example, age, daily life, profession, background etc.?	
•What kind of care did / do you receive during the pregnancy and how would you describe your experiences with this care?	
•Were you offered or did you receive any other care in addition to this, and how would you describe your experiences and needs with this offered or received care?	
•Is there any help or care you would have liked to receive but which was not offered to you?	
•Were or are you satisfied with the care you received and why?	

### Participants

We included women in vulnerable situations, meaning that these women were living in a potentially vulnerable situation at the time of the interview (e.g.,teenage pregnancies, living in poverty, housing problems). In addition, women had to be pregnant or had given birth in the year preceding the interview. Also, women had to live in the three northern provinces of the Netherlands (Friesland, Groningen, Drenthe). Finally, we aimed to recruit women with a varying number of children.

### Recruitment

A purposive sampling approach was used to obtain as varied a sample as possible in order to capture a wide range of experiences of women in vulnerable situations. Additionally, we used professional referral sampling [25], which means that we asked midwives and general practitioners to identify pregnant women in vulnerable situations who were offered or had received an additional intervention besides regular pregnancy care, and to ask these women whether they wanted to be interviewed by the researcher. With the consent of the women, these health professionals provided the research team with telephone numbers or email addresses of the women. We approached the women by phone (WhatsApp or SMS) or email to explain our intentions and ask whether they wanted to participate. We tried to approach these women shortly after their consent and stayed in contact with them in order to increase the chance of actual participation in the interviews.

### Research team characteristics

The research team consisted of three senior and two junior researchers, all women, from different professional and scientific backgrounds (health sciences, sociology, nursing, psychology and midwifery). These different backgrounds represent diverse research traditions and methods. Most of the researchers are from the north of the Netherlands and are familiar with the customs and habits of this region. Throughout the research team meetings, we reflected on the methodological choices that we made and the perspectives that we chose. This provided us with a deep and broad understanding of our research topic.

### Interview process

Interviews were conducted by MD during home visits or at another location chosen by the participant between October 2019 and December 2020. In all cases, only the woman, her child, and the interviewer were present during the interview. Due to the COVID-19 pandemic, we interviewed two women in video calls. The interviews started with a short introduction about the purpose of the study, and a few questions about the woman’s background. The topic list that was used was drawn up based on relevant literature, input from an advisory group of maternity health professionals, input from midwifery researchers, input from a lay expert, and input from a lay organization (Zorgbelang) (Table 1). We tested the topic list beforehand by conducting an interview with a young woman who had recently given birth but was not part of the target population. In addition, the list was modified as a result of discussions in the research team and with midwifery students. Throughout the iterative process of data collection, adjustments were made that allowed us to ask additional elaborative and supplementary questions to obtain more in-depth answers to the research question. Also, questions to validate results from previous interviews were added. The interviews were audio-recorded and transcribed verbatim. We invited every woman to read and check our script (member check). One woman wanted to do so, she did not have any corrections to the script.

### Analysis

A thematic approach was used to analyse the data throughout the research process [26]. First, the audio-recorded interviews were transcribed. Next, an initial familiarization took place through reading and rereading the transcripts. The first three transcripts were coded separately and independently. A codebook was prepared and discussed with several stakeholders (midwifery students, midwifery lecturers, researchers, and a lay organization (Zorgbelang)) which resulted in a refined codebook and adjustments to the topic list for the following interviews. Also, we asked input of a lay expert regarding the themes that were drawn from the codes. Data saturation was reached after 17 interviews as no additional themes or sub themes were identified. We used Atlas.ti (8.4) to analyse the data. Quotes were translated from Dutch to English by a translator.

### Ethics

The local Medical Research Ethics Committee of University Medical Center Groningen classified this study as non-WMO (Medical Research Involving Human Subjects Act, www.ccmo.nl) research (number 2019.259). Written informed consent was required before starting the interview. To achieve this, the researcher explained the meaning of informed consent during the initial contact with a participant. At the start of the interview, the researcher together with the participant read through the informed consent form before both signing it. To ensure confidentiality, personal data of the participants were separated from the transcripts and stored according to the data management rules of University Medical Center Groningen.

To minimise the risk of harm several steps were taken. Before the interview started the researcher repeated the purpose and design of the study to all women. Measures to ensure anonymity were also explained. Next, the researcher offered to answer any questions women might have, and also the researcher made sure women understood that withdrawal from the study was possible at any time.

## Results

### Recruitment process

A total of 22 women agreed to be contacted by the researcher in response to the invitations from the health professionals. Five women withdrew their participation. Some indicated why they wanted to withdraw their participation, others withdrew at the last minute by sending a message or by not showing up at the chosen location and not responding to messages. Even after women agreed to participate, staying in touch with women remained a challenge. The researcher sensed feelings of distrust, detachment, and hesitation among the women before and during the interviews. It sometimes took multiple reassurances, for instance about “not having to tell everything that happened to them again,” to make them feel comfortable enough to answer the interview questions. Our final sample consisted of 17 women. If necessary, women had the opportunity to contact the researcher after the interview. None of the women made use of this. The interviews ranged from 19 to 50 minutes, with an average duration of 35 minutes.

### Background characteristics

All women lived in the three northern provinces of the Netherlands (Table 2). Their mean age was 22 years. Most of them had already given birth. Seven women did not start or complete a vocational education. One woman with a high level of education (university level) was included. She could be considered vulnerable because circumstances caused her to lose her house, which left her unemployed and in need of help to find accommodation. Four women were unable to work due to physical (e.g. pelvic girdle pain) or psychological issues (e.g. compulsive disorders or psychosis). In addition to standard prenatal care, all women received additional maternity care interventions because of their vulnerable situation, such as being young and/or single, dealing with psychological or relational issues (some with concerns about their safety), and often having socioeconomic issues. Past addictions, previous contact with ‘youth care’, and being intellectually impaired were also mentioned.

**Table 2:** Background characteristics of the participants (*N* = 17)

	n
**Mean age**	22, range 19–37
**Pregnant during interview**	6
**Given birth to:**	
First child	6
Second child	4
Fourth child	1
**Province:**	
Groningen	6
Friesland	3
Drenthe	8
**Living situation**	
Single	4
With partner	13
**Completed vocational education**	
None	7
First level (assistant training)	1
Second level (basic vocational training)	3
Third level (professional training)	5
High level of education (university level)	1

**Fig. 1:**
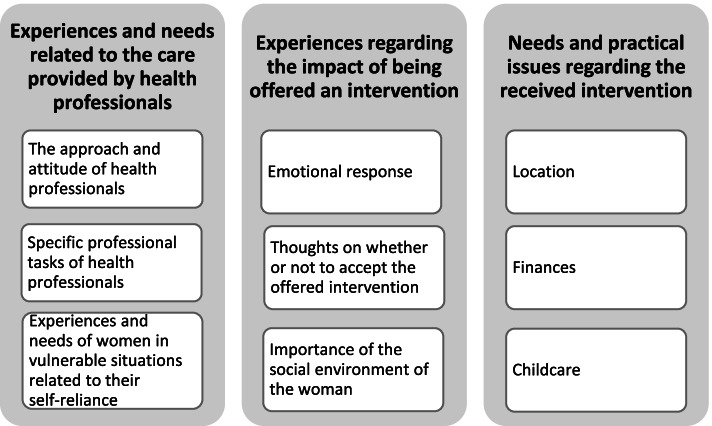
‘Experiences and needs of women in vulnerable situations who receive additional maternity care interventions’, themes and underlying categories

The experiences and needs of women in vulnerable situations who receive additional interventions in maternity care can be summarized in three overarching themes with underlying categories: experiences and needs relating to the care provided by health professionals, experiences regarding the impact of being offered an intervention, and needs and practical issues regarding the received intervention (Fig. 1). Every category has the potential to facilitate or to hinder participation in an intervention. Up to interview 14, if new information came to light for which there was not yet a category, we constructed a new category. In interview 15 and 16 we only added information to the already existing categories. Interview 17 did not provide any new information.

### Experiences and needs related to the care provided by health professionals

Women reported many experiences and needs related to the care provided by the health professionals. We categorized these experiences and needs into ‘the approach and attitude of health professionals’, ‘specific professional tasks of health professionals’, and ‘experiences and needs of women in vulnerable situations related to their self-reliance’.

#### The approach and attitude of health professionals

Participants felt that they were sometimes approached with prejudice.

“That’s a really weird line of reasoning, you can’t just argue that ‘you’re young, you are not in a relationship, so that’s that: abortion’. That’s not how it works. It’s my body, I get to decide that.” W16.

Women wish to be taken seriously and treated without prejudice, with respect and dignity, as valuable, equal individuals. These women’s experiences of being stigmatized by people in general and by health professionals evoked feelings of anxiety and distrust.

“And that’s when the rage came bubbling up, like it’s so unfair that I am being placed in a box and treated like this based on my past. I come here for help and it feels like I’m really just torn down.” W16.

This was a reason for women to hesitate to accept care, to report private issues, or even to reject the care offered.

“That is an effect insofar as that you feel uncomfortable, and you will make a call less quickly when something’s genuinely wrong, because you’re already certain that people simply won’t listen to you. You really know that in advance already, before you call: ‘Well, they won’t listen anyway’.”W6.

We also found that a positive approach of the health professional to women in vulnerable situations was an important need. Women indicated that health professionals should take sufficient time when offering or considering offering additional care to them. There should be a genuine intention to meet the needs and wishes of women. Women liked health professionals who are easily accessible and approachable. Personal traits such as “friendly”, “open”, and “straightforward” were often mentioned. These personal traits could persuade women to participate in an intervention.

“She was really compassionate, she really wanted to help me from the bottom of her heart … and really ask how you’re doing and whether they could really do something for you, you know.” W9.

#### Specific professional tasks of health professionals

The women stated that counselling of health professionals on the additional intervention did not always correspond to their needs. They stated that they needed health professionals to provide honest information that was customized for them. Women felt overwhelmed by the amount of information given to them.

“So, I THINK that by giving too much information, that you can make people more frightened than necessary. Look, it can work really well for some people and there are people who can handle a lot of information really well, but I can’t.” W3.

Standardized counselling also did not meet the needs of some of the women. In addition, women had the impression that information about an intervention was selectively shared by health professionals, and that the health professional decided which information was appropriate for a woman.

“And that’s what I’m afraid of in Youth Care. They will say: you are now registered with Youth Care and you will now follow our path, while I am like, I want it to go the way I want it. That is what I am afraid of in Youth Care. I do not want that at all.” W8.

Women searched for the needed information themselves if the information and/or counselling provided did not meet their needs. Information was mainly provided verbally, sometimes accompanied by brochures or by a form to fill in. The women stated that they considered a combination of both to be useful.

In addition, the women indicated a desire to remain in control of their own care. They stated that if health professionals initiated actions on their behalf, they wanted to be kept informed of these actions (e.g. by making certain arrangements or contacting other professionals to get things done). Moreover, the women expressed a desire for continuity of care. Experiencing a sense of continuity of care meant a lot to the women. They mentioned that health professionals had to take responsibility for ensuring that every health professional involved in their care was up to date, well informed, and on the same page.

“Because at some point it becomes confusing as to who knows what. Because then the gynecologist comes or the district nurse, who says, she says, like: ‘Yeah, you have to do it this way or that way’. Yes, I already heard that from the other coach, so I don’t need to hear that again.” W10.

The women perceived shortcomings in this regard. For example, they felt compelled to tell their story over and over again to various other health professionals involved in their care.

“And you’re being sent home with the comment: ‘Hey, if you’re not doing well, you should call again’. You basically have to repeat your story constantly. Constantly. Nothing is written down and no issues are put through to others. You’re constantly trapped in that story and you’re constantly explaining yourself.” W6.

The women also wanted the interventions offered to be related to the circumstances they were facing. Circumstances mentioned were the need for socioeconomic support to help overcome a financially unstable situation, or psychological or relational issues. According to the women, health professionals should adjust the care to meet the specific, individual needs of women, i.e. the right care at the right time, in the right amount. After agreeing to participate in an intervention, the women wanted the intervention to start quickly. The fulfilment of these specific needs was of great importance to the women.

“That’s very important to me, because I want to know how things go and how they happen and how are you going to arrange things. Unfortunately, it turns out, we’re nearly a year into this – over a year, really. And we’ve only had the right care for three months.” W13.

#### Experiences and needs of women in vulnerable situations related to their self-reliance

Having freedom of choice in accepting or declining an intervention was important for the women. The women desired a sense of self-determination and autonomy and wanted to be involved in decisions about the provided care, both in terms of content and in its course. Having the freedom to choose resulted in motivation to accept the intervention and caused the women to experience a sense of trust and reassurance. Women also expressed their ambition to learn.

“That they might have said something like: ‘Can we just do this together’. I had already indicated several times during (her) conversation that it wasn’t working that well and that kind of thing. I don’t need them to spell everything out for me, because you don’t learn anything that way.” W4.

Sometimes, a sense of independence made the women reluctant to accept an intervention or to ask for help, because they imposed standards on themselves by thinking that they should be able to cope with their circumstances by themselves.

“Yes, I’m really very independent and that’s also my pitfall. I know that I need help, but I won’t ask for it easily. Then I’m like, I’ll just muddle through until I really need something. That’s how I was raised, that you carry on, carry on, carry on. And only later on, now, in the last few years, I have learned a lot.” W14.

### Experiences regarding the impact of being offered an intervention

The offer of an additional intervention had an impact on the women. They expressed a variety of feelings and reactions towards this offer. We divided these reactions into ‘emotional response’, ‘thoughts on whether or not to accept the offered intervention’, and ‘importance of the social environment of the woman’.

#### Emotional response

The women said that the initial offer of an additional intervention came as a shock to them and triggered feelings such as anxiety, distrust, and protectiveness towards their (unborn) child.

“Well, I felt confronted by that. And I was scared that they would think that I might not be able to handle this and that my child might then be taken away from me. And that I would be monitored completely. That was my first fear.” W3.

Fear of having their child taken from them and getting stuck in a trajectory of care (of ‘Youth care’) were mentioned.

“At first I was like, yeah, fine, you know. I have nothing to hide. But that does slowly change into the feeling of, gosh, it better not be like one person gets to have a conversation with me to see if I can financially support my child. If they think that I tick the ‘no’ box, that I have to give up my baby.” W13.

As soon as the women participated in an intervention, feelings of anxiety, chaos, and confusion reappeared, mainly due to the number of professionals who interfered in their situation, and the perceptions that health professionals had of their situation.

In some cases, the women reacted positively when they were invited to participate in an additional intervention because they were open to the help offered. Participation in an intervention gave these women a sense of reassurance and of being acknowledged.

“It just feels a lot more certain, because I know how I want things to be and how I want to do it. But to achieve that goal, it’s good to have someone behind you who says you’re doing well.” W5.

Moreover, it gave them an opportunity to properly prepare for their future family life. Women described these effects as a boost to their self-esteem. Furthermore, actions of health professionals to make necessary arrangements for them in relation to, for example, financial issues, childcare, or accommodation gave the women a sense of inner peace. They mentioned that participating in the intervention had a beneficial and de-stressing effect on the stress caused by rules and regulations, including those imposed by the government.

*“*… , but also, for example, some more peace while sorting things out, the day care, and, well, more things that you have to sort out. But also, just my own paper work, questions that I had, that gives you a little extra peace.” W14.

#### Thoughts on whether or not to accept the offered intervention

Women shared their thoughts on deciding whether or not to accept the offered intervention. On the one hand, they mentioned that they themselves had to be open to the additional help offered. In their opinion, the extent to which they were open to care was influenced by their personality and age, and by the responsibilities of being a future parent. The women said that their pregnancies acted like catalysts to positively accept the intervention and that participating in an intervention prepared them for the best possible pregnancy, delivery, and future family life.

“Because now I will literally do anything for my child … How do I put this? It’s a little child, it’s fragile, it’s completely dependent etc. So everything has to be provided by mum. Yes, or by dad, so you have to make the choices for your child.” W15.

On the other hand, women accepted the intervention as a negative or conditional choice because participation gave them access to the fulfilments of their needs. For instance, by accepting to participate in an intervention, they gained a financial benefit or they were given the opportunity to negotiate about special other care.

“But I also think that, because they give me 50 euros every Monday … So, sometimes it happened that I really didn’t have any money, but I did have those 50 euros. This meant that I could eat. You see? Get food for my child and stuff. So, yes, I’m happy about that. Yes, I did have that.” W2.

#### Importance of the social environment of the woman

The women stated that their spouse, family, or friends played an important role in whether they wanted to accept the offered intervention. This influence could have moved them to decide to accept the offer. For instance, one woman mentioned the support of her grandparents, who accompanied her on all visits to health care providers. Furthermore, a woman stated that the presence and support of family gave her some peace of mind.

“If there really are other things going on, or private matters that I’d rather not discuss with them, then I always have friends who already have kids or who understand these things. I’d sooner do that, because I am a bit of a private person. So, if there really is an issue, then I’ll just text someone or I visit someone in my own circle.” W16.

A lack of social support from close ones was also expressed, which could have had a negative impact on accepting an offering or attending an already accepted intervention.

“I honestly say I have not been there twice [Centering Pregnancy meeting], during those meetings I had to bring my partner and he did not want to come. W8”.

### Needs and practical issues regarding the received intervention

The needs of women in vulnerable situations regarding additional interventions included several practical issues. We categorized these issues into ‘location’, ‘finances’, and ‘childcare’.

#### Location

The location and its atmosphere were important for the women.

“Um, well, I do think that it matters where, where the location is, you know, because if it’s difficult for you to take the step, you know, and you have to travel quite far to get there, that makes the step more difficult to take.” W4.

Many women preferred the intervention to take place in their homes, as this was practical with regard to preparing for the future baby. The environment of their homes signified safety and comfort to them. When the intervention took place elsewhere, the availability of transport to the location of the intervention influenced the acceptance of an intervention.

“Yes, that does play a part. Because it has to do with transport, how can I get there? It also has to do with burden, because an appointment, that means you have to get there and get back home again. That actually is a burden, that certainly played a part for me.” W13.

Women wanted to be familiar with this location. The location had to be practical, i.e. accessible, easy to find, and in a safe neighbourhood. Furthermore, the atmosphere of the location (e.g. a health centre) was important for the women, but we found a wide variation in wishes: for some, the location should not come across as ‘too clinical or sterile’, while others wanted a quiet, peaceful location so as not to be overwhelmed and over-stimulated.

#### Finances

The women stated that the concept of money, such as having to pay for the intervention or not, may have influenced their decision to accept or continue and complete the offered maternity care interventions. Some of them simply had no money to spend due to debt. Others wanted to weigh up the expected benefits, advantages, and costs of the offered intervention before accepting.

“Um, well, then I would start having doubts, like, at the time they offered it to me, you know, that they said yes, you can get guidance, but you have to make a financial contribution, then I would have thought longer like ‘OK, do I want this or not?’ That would have increased the chance of me saying no. At least, that was the case for everything, and it still is when they offer me something, or anything, you have to make a contribution, then I think ‘Is this useful?” W1.

Others claimed that having to pay for their care would have made no difference to them, as they were convinced of the necessity and advantages of the intervention.

#### Childcare

The women indicated that having to care for another child could be a practical reason for accepting or declining an intervention. Some women stated that, if necessary, their spouse, family, or friends would be willing and able to babysit so that the woman could participate in an intervention. For others, childcare could not always be arranged. One woman stated that she felt such maternal responsibility for her child that she found it psychologically difficult to leave her child with someone else in order to attend an intervention.

“Well, see, my mother lives here and we were six at home. One sister lives in Arnhem, but the rest lives here. And I’m lucky to have them, because I can ask them to babysit sometimes and stuff like that. Otherwise, it all comes down to me. And that’s difficult sometimes, because my son, at the time, I had to take him everywhere. And that didn’t work very well for me. Well, how do you visit a mental health care facility if you have to care for your child?” W14.

## Discussion

The aim of this study was to gain insight into the experiences and needs of women in vulnerable situations who receive additional maternity care interventions in the Northern Netherlands. The 17 individual semi-structured interviews showed that living in vulnerable situations and receiving an offer for additional care evoked a range of reactions and emotions from pregnant women. In general, we found that women who lived in vulnerable situations and who were pregnant wanted the best possible pregnancy, delivery, and future family life. All women in our study accepted the offer and stated that the received intervention was valuable to them. We identified three themes, namely experiences and needs related to the additional care provided by health professionals, experiences related to the impact of being offered an additional intervention, and needs and practical issues related to the offer. Within these themes, we identified facilitating and impeding factors. We found that the needs of pregnant women in vulnerable situations who received an additional maternity care intervention varied. This variation in needs was mainly related to practical issues. Women also expressed common needs, namely the desire to have control over their situation, the wish to receive tailor-made information about the intervention, and the wish for the intervention to be specifically tailored to their circumstances.

In our study, women emphasized their desire for self-determination, to learn and to be and become autonomous. To truly involve women in their own care, interpersonal relationships can empower women to make their own decisions [27]. A mutually respectful and attentive relationship between the health professional and the woman should be the starting point for jointly ‘designing’ an intervention that fits her individual and unique needs and preferences. Within this relationship, respectful interaction is important in order to prevent the creation of barriers and to perpetuate vulnerability [28].

We found that women experienced anxiety and fear of stigmatization when and after they were offered an additional intervention [19]. In a Dutch study that examined the perceived socioeconomic position-related stigma among people with low SES, it was found that people’s reactions were related to feelings of inferiority, being physically recognizable as a poor person, being responsible for their own financial issues, and experiencing feelings of shame [29]. Shame is a complex phenomenon and can interfere with the process of gaining self-esteem, which is required to be and become autonomous [30]. Therefore, health professionals should be aware of women’s feelings of fear and anxiety as well as their own perception of women and their issues, in order to prevent the emergence of barriers that increase the vulnerability of pregnant women. Stigmatizing attitudes of health professionals negatively affect the provision of care and could lead to women not wanting to start an intervention or interrupting it during the care process [29].

Women expressed their desire and need for continuity of care. They perceived problems related to a lack of continuity within the additional care that they received. It is known that continuity of care yields better results for mothers and their babies [31]. Especially for people in (vulnerable) situations whose care involves a large number of health professionals, continuity of care can be very important, both to help manage and plan the additional care needed and to develop a trusting and empowering relationship with the women [32].

Many interventions have been developed with the aim of supporting women to help make their situation less vulnerable [23]. However, health professionals should think carefully about which interventions they offer. Effects of interventions are not always proven and may even lead to unintended harm. Allen-Scott et al. (2014) explained this in their scoping review in which they emphasize that when an intervention is implemented, there must always be awareness on what the effects and unintended effects may be in order to prevent harm to women psychologically, somatically, psychosocially and culturally [20].

### Strengths and limitations

A major strength of our study is the number of women we interviewed. With 17 interviews we reached data saturation and indexed the experiences and needs of women in vulnerable situations with additional maternity care interventions. The accumulated results are representative for the northern region of the Netherlands.

This study also has limitations. All women in our study participated in an intervention, and all women in our study recognized their own need for help. We have not talked to women who have turned down such an offer and therefore do not know why they did so. It is possible that women who refuse to participate in an intervention are more likely not to recognize their needs and therefore have other experiences and needs regarding the offer of an additional intervention during their pregnancy. However, it must be taken into account that women may also have other reasons. In addition, in response to our offer to read and check the interview script, most of the women indicated that this was not necessary because they stood by what they had told us, so no member check was performed.

### Recommendations

Within education and practice we recommend that more attention is paid to developing social empathy for groups in vulnerable situations in students and health professionals. Segal et al. (page 131, 2013) defined social empathy as “the ability to understand people by perceiving or experiencing their life situations and therefore gain insight into structural inequalities and disparities” [33]. Developing social empathy can help students and health professionals identify the impact of their actions on groups of people in vulnerable situations [34]. In addition, we recommend structurally involving pregnant women in vulnerable situations in policy-making. Experts by experience can be of great importance in teaching students and health professionals how to raise awareness about the difficulties that women in vulnerable situations experience in their daily lives, resulting in the production of tailor-made interventions for women in vulnerable situations [20].

Throughout this study, the issue of stigma and stigmatization seemed to be a factor for women. Stigmatization does indeed influence the way in which women experience and take decisions about additional interventions. The background of stigma in this specific context of pregnant women in vulnerable situations is not yet fully known. Further research is required to better understand the background of stigma, and how these stereotypes and misconceptions can be overcome. Furthermore, our results can be used in further studies with quantitative designs, e.g. to validate our results in larger populations or populations in other regions, and with qualitative designs by considering and discussing them within communities of health professionals and women to translate them into practical recommendations for improvements. Finally, we recommend exploring how healthcare for pregnant women in vulnerable situations can be considered from a relationship-oriented perspective in which the needs and wishes of women are aligned with the skills and needs of health professionals. Within this care, health professionals behave in an empathetic, sincere, inclusive, and reciprocal manner that stimulates self-management and self-reliance of the client [35].

## Conclusions

This study provides insight into the experiences and needs of women in vulnerable situations with regard to additional maternity care interventions in the north of the Netherlands. We highlight that in order to tailor care to the individual perspectives, needs, and circumstances of women in vulnerable situations, health professionals must be or become aware of their own attitudes and prejudices towards these pregnant women. They need to invest in building empathetic and mutually respectful relationships with women, so that women in vulnerable situations become more empowered, autonomous, and self-sustainable. Continuity of care is of great importance and can act as a catalyst for better outcomes for both mother and child.

## Data Availability

The datasets used and/or analysed during the current study are available from the corresponding author on reasonable request. However, we should note that the data are written in Dutch.
